# Somatic BRCA Mutation in Metastatic Breast Cancer

**DOI:** 10.7759/cureus.49600

**Published:** 2023-11-28

**Authors:** Tristan B Minick, Robert A Norman

**Affiliations:** 1 Dermatology, University of Florida, Gainesville, USA; 2 Dermatology, Nova Southeastern University Dr. Kiran C. Patel College Of Osteopathic Medicine, Fort Lauderdale, USA

**Keywords:** wide-excision, cancer genomics, skin lesion biopsy, breast cancer metastasis, brca gene mutation

## Abstract

A 65-year-old female with a history of multicentric invasive ductal breast carcinoma with lobular features and prior mastectomy presented with a chief complaint of two new raised mildly erythematous lesions on the right upper arm. The lesions were visualized during examination, and the patient noted no symptoms associated with them. Tangential shave biopsies were obtained for each lesion and were sent to the lab for testing. Both lesions were found to be metastatic breast carcinoma. Wide local excisions were performed on each site. The patient followed up with radiation therapy and was prescribed Faslodex and Ibrance. FoundationOne testing on the lesions revealed BRCA2 loss in the tumor, and germline DNA testing was performed in light of this. The test yielded negative results for harmful BRCA1 and 2 mutations. The patient was treated with Lynparza (olaparib), and two years following the start of this medication has had no additional recurrences.

## Introduction

Breast cancer makes up around 30% of all cancer cases in women, and up to 10% of these cases are hereditary. The other 90% of breast cancers are the result of either somatic genetic or epigenetic alterations [1]. Two of the most frequent culprits that cause hereditary breast cancer are known as the BRCA1 and BRCA2 genes. Around 72% of women who are BRCA1 carriers and 69% of women who are BRCA2 carriers will develop breast cancer by the age of 80. In contrast, about 13% of women in the general population will develop breast cancer during their lifetime [2,3]. 

The BRCA1 and BRCA2 tumor suppressor genes play a major role in homologous recombination, a vital process for repairing double-stranded DNA breaks. When cells do not have functioning copies of these genes, DNA repair must be done through other more error-prone methods that can lead to critical mutations. While this usually leads to the death of the cell, it can sometimes generate a cell capable of autonomous cell division and metastasis, the hallmarks of cancer [4]. These harmful mutations, known as a germline mutation, are most commonly inherited from an individual's parents. Alternatively, the mutation can develop due to genetic and environmental factors that are not inherited, known as a somatic mutation. Some therapeutics are available that can target the DNA repair process to prevent the formation of cancerous cells from cells that have mutated BRCA genes.

## Case presentation

The patient presented initially with locally advanced multicentric invasive ductal carcinoma of the right breast. The tumor was found to be positive for estrogen receptor (ER) and progesterone receptor (PR) and negative for HER2/neu with high Ki-67. She underwent neoadjuvant chemotherapy with dose-dense AC x4 followed by taxane x4. She also had a bilateral skin- and nipple-sparing mastectomy followed by tissue expander reconstruction. Following these treatments, pathology showed a near-complete response in the breast and lymph nodes. She began taking Arimidex, followed by Aromasin.

**Figure 1:**
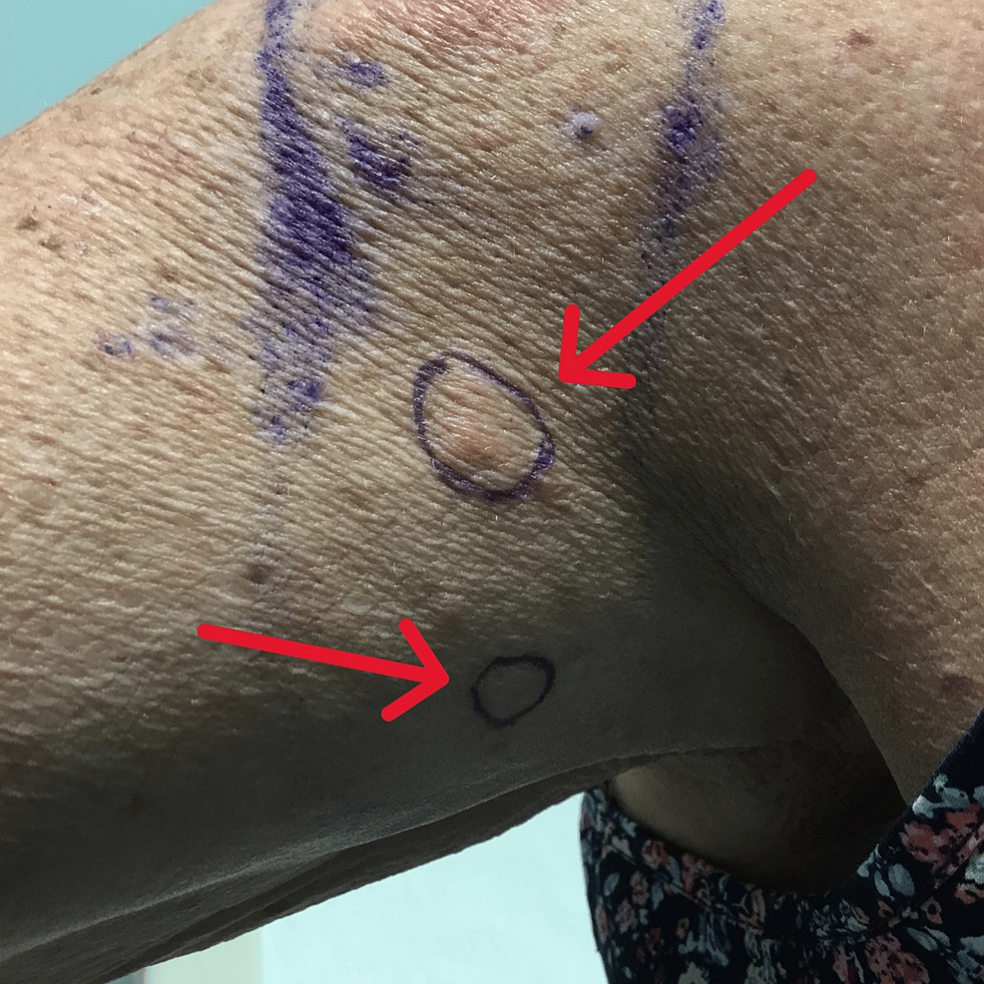
Neoplasms on the upper right arm Two raised mildly erythematous papules on the patient's upper right arm.

**Figure 2:**
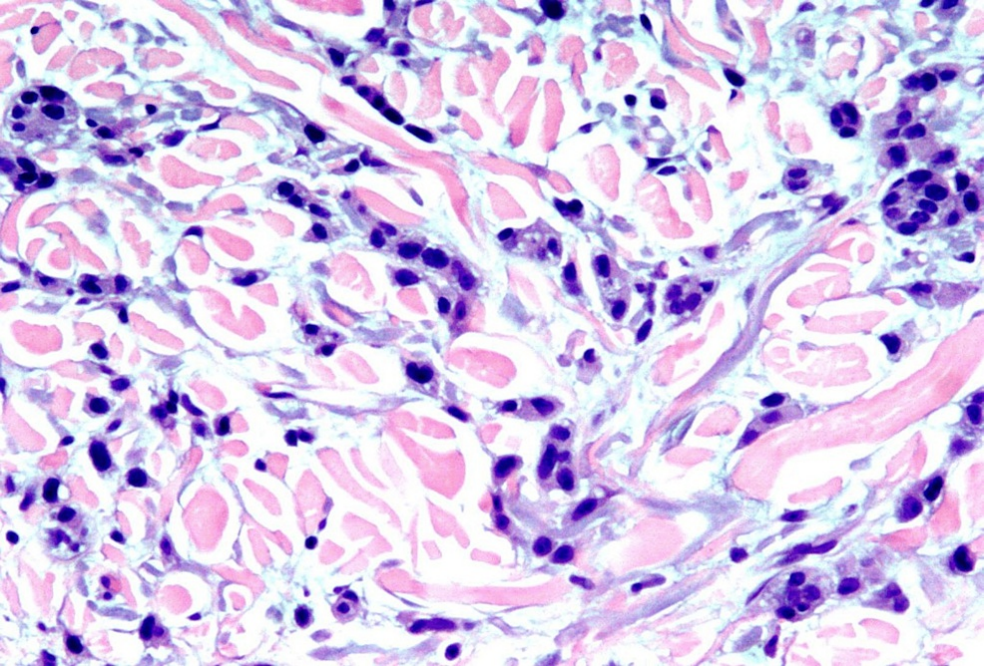
Biopsy of arm lesion Atypical infiltrate of tumor cells with a high nuclear-to-cytoplasm ratio in the reticular dermis is identified. The tumor cells are somewhat nevoid and distributed as small nests, solitary cells, and cords.

Mildly erythematous papules were raised on the patient's upper arm (Figure 1). A shave biopsy measuring 0.5 x 0.5 x 0.1 cm was obtained from the inferior papule. Another shave biopsy measuring 0.6 x 0.5 x 0.1 cm was obtained from the superior papule. The biopsies were sent to the lab to rule out metastatic breast cancer vs. squamous cell carcinoma and basal cell carcinoma. The biopsies showed that both lesions were consistent with metastatic breast carcinoma, with the tumor cells in each sample being positive for cytokeratin 7 (CK7) and ER/PR and negative for HER2 and melanoma-associated antigen recognized by T cells (Mart-1) (Figure 2).

After discovering this, the patient was taken off the Arimidex and Aromasin. The patient had a PET/CT scan and MRI of the breast and right shoulder, none of which showed evidence of disease. The patient underwent a wide local excision with no evidence of gross disease remaining. The procedure showed metastatic carcinoma compatible with breast primary extending to inked superior, lateral, and deep margins, with additional atypical melanocytic proliferation identified appearing to focally involve the peripheral margins of excision. She completed radiation therapy and was prescribed Faslodex and Ibrance. She was also using Silvadene at the site of radiation burns on her right upper extremity.

Eight months later, it was found that she had a progression of the disease in the bones, and she was taken off Faslodex and Ibrance. She underwent palliative radiation therapy to the right shoulder bone and began letrozole and Afinitor but had tolerance issues with these medications. Another PET/CT scan four months later showed stable findings, but tumor markers were rising, and she had recurrent hypercalcemia and anemia, so treatment was held due to her worsening condition.

FoundationOne testing was completed on the tumor recurrence from her arm, which revealed microsatellite status stable with intermediate tumor mutation burden with 8MUTS/MB. Additionally, it revealed ASXL1 alteration, CDH1 alteration, MML2 alteration, RB1 loss, TET2 alteration, PD-L1 is 0% negative, and BRCA2 loss. In response to the discovered BRCA2 loss, germline DNA testing was completed to check for harmful BRCA1 and BRCA2 mutations. This testing came back negative for both BRCA1 and BRCA2. In light of the tumor being found to have BRCA2 loss, the patient was started on Lynparza, and since then, her tumor markers have remained normal. A follow-up PET/CT scan two years after starting Lynparza showed no change from the previous scan with no active disease.

## Discussion

Here, we present a case of a 65-year-old female with a history of multicentric invasive ductal breast carcinoma who had two recurrences of metastatic breast carcinoma on her right upper arm. The presence of multiple foci of breast cancer in breast cancer specimens has been shown to be anywhere from 9-75% depending on multiple factors, including the definitions that are used, the mode of detection, and the pathological assessment. The survival rates of multicentric invasive ductal breast carcinoma vs unifocal breast cancer post total or segmental mastectomy haven't been found to be significantly different in randomized clinical trials [5].

The patient tested negative for BRCA1 and BRCA2 germline mutations, but her tumors were found to have BRCA2 loss. The BRCA gene is responsible for double-stranded break repairs in our DNA, and any mutations present in the gene have the potential to cause a loss of function that results in further mutations during cell growth. It is common for women to undergo germline DNA testing to determine if they have a harmful BRCA1 or BRCA2 mutation that may lead to them developing breast or ovarian cancer later in their life. Somatic testing of the tumor, however, is not as common.

There are alternative treatment options for breast cancers that are caused by germline BRCA mutations, such as poly-ADP ribose polymerase (PARP) inhibitors [6-8], but in patients like the one presented here, simple germline testing would not provide an indication that these treatments should be considered. In patients who present with breast carcinomas, it is important to consider alternative testing methods, including somatic testing of the tumors, to determine the best treatment plan for each patient. This somatic testing allowed the patient to be treated with the PARP inhibitor olaparib.

PARP inhibitors work by stopping PARP enzymatic activity, which prevents the PARP protein from helping repair single-stranded breaks in the DNA. This then leads to an increase in double-stranded breaks (DSB), which need to be repaired, which is normally the job of the BRCA genes. When a tumor has two copies of non-functioning BRCA genes, the cells cannot repair these breaks, leading to cell death. In patients with germline BRCA mutations, their somatic cells typically have one functioning and one non-functioning copy of the gene, while their tumor cells have two non-functioning copies. This makes them the perfect candidate for the use of PARP inhibitors since the tumor cells won't be able to repair the DSBs and will die, while the somatic cells with one functioning copy of the BRCA gene will still be able to repair the extra breaks and will survive [9]. In patients like ours, the same basic principle applies. The BRCA2 loss that was found in the tumor but not in the somatic cells means that a PARP inhibitor will be able to selectively kill the tumor cells while leaving the rest of her cells unharmed.

## Conclusions

Here, we presented an unusual case of metastatic breast cancer developing a somatic BRCA mutation that would've gone undetected without comprehensive DNA testing. This case displays the importance of a multidisciplinary approach to treating lesions like the ones presented in this report to ensure that the most accurate diagnosis allows for the most effective treatment. This case may also support the use of PARP inhibitors in treating cancers with somatic BRCA mutations rather than the typical germline mutations. Further research into this topic is necessary to determine the best course of action for detecting and treating these uncommon mutations in breast cancer.
